# Tiny Microbes with a Big Impact: The Role of Cyanobacteria and Their Metabolites in Shaping Our Future

**DOI:** 10.3390/md14050097

**Published:** 2016-05-17

**Authors:** Sophie Mazard, Anahit Penesyan, Martin Ostrowski, Ian T. Paulsen, Suhelen Egan

**Affiliations:** 1 Department of Chemistry and Biomolecular Sciences, Macquarie University, Sydney NSW 2109, Australia; sophie.mazard@mq.edu.au (S.M.); anahit.penesyan@mq.edu.au (A.P.); martin.ostrowski@mq.edu.au (M.O.); 2 Centre for Marine Bio-Innovation and School of Biological Earth and Environmental Sciences, University of New South Wales, Sydney NSW 2052, Australia; s.egan@unsw.edu.au

**Keywords:** natural products, microalgae, biotechnology

## Abstract

Cyanobacteria are among the first microorganisms to have inhabited the Earth. Throughout the last few billion years, they have played a major role in shaping the Earth as the planet we live in, and they continue to play a significant role in our everyday lives. Besides being an essential source of atmospheric oxygen, marine cyanobacteria are prolific secondary metabolite producers, often despite the exceptionally small genomes. Secondary metabolites produced by these organisms are diverse and complex; these include compounds, such as pigments and fluorescent dyes, as well as biologically-active compounds with a particular interest for the pharmaceutical industry. Cyanobacteria are currently regarded as an important source of nutrients and biofuels and form an integral part of novel innovative energy-efficient designs. Being autotrophic organisms, cyanobacteria are well suited for large-scale biotechnological applications due to the low requirements for organic nutrients. Recent advances in molecular biology techniques have considerably enhanced the potential for industries to optimize the production of cyanobacteria secondary metabolites with desired functions. This manuscript reviews the environmental role of marine cyanobacteria with a particular focus on their secondary metabolites and discusses current and future developments in both the production of desired cyanobacterial metabolites and their potential uses in future innovative projects.

## 1. Introduction

Cyanobacteria are photosynthetic prokaryotes. Despite the fact they are often referred to as blue-green algae, they have no direct relation to higher algae. They are believed to be one of the oldest organisms on Earth with fossil records dating back 3.5 billion years [1,2]. Cyanobacteria are responsible for the Earth’s transition from a carbon dioxide-rich atmosphere to the present relatively oxygen-rich atmosphere as a consequence of oxygenic photosynthesis [3]. Throughout their long evolutionary history, cyanobacteria have diversified into a variety of species with various morphologies and niche habitats.

Cyanobacteria present a diverse range of morph types, including unicellular, surface-attached, filamentous colony- and mat-forming species. Several species form important symbiotic associations with other micro- and macro-eukaryotes [4,5]. In keeping with the broad taxonomic diversity across the phylum, cyanobacteria inhabit a diverse range of terrestrial and aquatic habitats, ranging from deserts to freshwater and marine systems across a range of eutrophic and oligotrophic conditions. They can also be found in extreme environments, such as Antarctic dry valleys, Arctic and thermophilic lakes [6,7], as well as in unlikely habitats for phototrophs, such as in the subsurface of calcareous rocks (*Gloeobacter violaceus*) [8] and Lava Caves [9].

Throughout their evolutionary history, cyanobacteria have developed unique interactions with other (micro- and macro-) organisms. Many of these interactions are based on a multitude of unique and complex genetic pathways leading to the production of secondary metabolites [4,5]. Secondary metabolites from cyanobacteria have been studied traditionally for their involvement in disease, e.g., microcystins and cylindrospermopsin, which trigger gastrointestinal illness, liver disease and kidney damage, or for their medicinal properties, such as anticancer, antimicrobial and UV-protective activities. The last decade has seen an increased interest in cyanobacterial research, resulting in an expansion of the uses of cyanobacterial metabolites beyond the realms of public health and pharmaceutical industries to include pigments, food and fuel production and other biotechnological applications [10,11].

Several recent publications have extensively reviewed the diversity and genetics of secondary metabolite production in (marine) cyanobacteria [12,13,14,15]. Therefore, here, we summarize this information and present insights into the current transition of research from traditional chemistry-based screens to molecular engineering and synthetic biology. These advances will not only contribute to basic knowledge, but will also further drive the use of cyanobacterial secondary metabolites in novel applications.

## 2. Environmental Impact of Marine Cyanobacterial Secondary Metabolites

**Figure 1:**
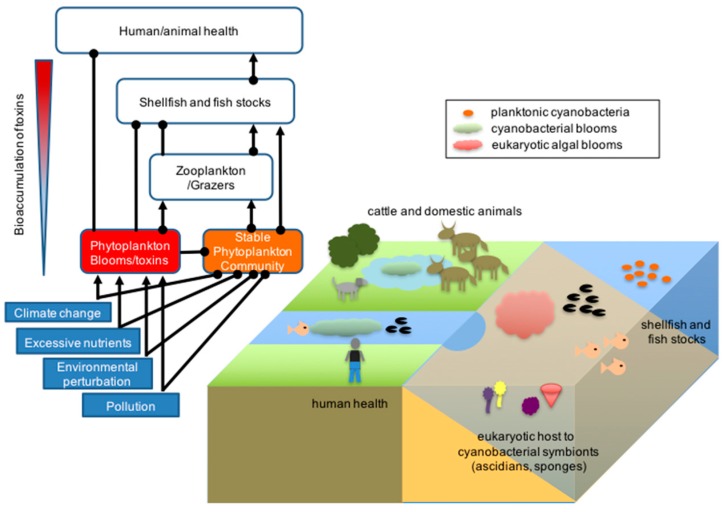
Environmental impact of photosynthetic microorganisms in aquatic systems. Different classes of photosynthetic microorganisms are found in aquatic and marine environments where they form the base of healthy food webs and participate in symbioses with other organisms. However, shifting environmental conditions can result in community dysbiosis, where the growth of opportunistic species can lead to harmful blooms and toxin production with negative consequences to human health, livestock and fish stocks. Positive interactions are indicated by arrows; negative interactions are indicated by closed circles on the ecological model.

Some of the earliest research on cyanobacterial secondary metabolites derived from the study of toxins produced by harmful algal blooms (HAB) and was mainly focused on freshwater species [16,17,18]. Toxin production by HAB can have dramatic health and economic impacts in lakes, rivers, estuarine and coastal shores, resulting in the death of cattle and domestic animals, as well as shellfish poisoning, leading to substantial financial loss to industries (Figure 1) [19].

The structure, cellular target and bioactivity of HAB toxins are broad and include soluble compounds of several types, such as neurotoxins, hepatoxins, cytotoxins, dermatoxins, in addition to endotoxins, e.g., lipopolysaccharides (LPS). The best-studied examples of cyanobacterial toxins are the neurotoxins; anatoxin-a/saxitoxin (*Anabaena flos aquae*) [20,21] and the potent hepatotoxin microcystins (*Microcystis* sp.) [22]. However, while some of these toxin-producing freshwater cyanobacterial species can expand into estuarine environments, it is interesting to note that toxin-producing unicellular species rarely predominate in truly marine habitats [18]. In the marine environment, toxin production appears limited to the filamentous colony-forming cyanobacteria, Oscillatoriales, *Trichodesmium, Lyngbya* (reclassified as *Moorea* sp. [23]) and *Nodularia*, and the (phyto) planktonic dinoflagellates and diatoms. Indeed, similar to freshwater cyanobacteria, these species form recurrent seasonal outbreaks leading to toxic blooms affecting shellfish and finfish stocks with dramatic consequences for aquaculture and human consumers [24]. The greater occurrence of HABs in estuarine and coastal waters has been linked to increased eutrophication, in particular nitrogen and phosphorus loading due to runoff from agricultural land. In recent times, greater public awareness and better agricultural management practices in many developed countries have reduced the occurrence of nutrient-induced HABs. However, ecosystem perturbations, such as localized heat waves, and habitat stress from human activities, including aquaculture, urbanisation and shipping, are increasingly linked to recurrent HABs [17,25], potentially as a result of the dysbiosis of microbial communities that form the base of healthy marine ecosystems.

Several marine cyanobacteria produce toxins, although these genera appear less prevalent in oceanic compared to coastal settings. Marine cyanobacterial blooms are more prominent in tropical and sub-tropical regions, mainly in shallow reef areas. The main bloom-forming species include *Synechocystis*, *Oscillatoria*, *Lyngbya* (*Moorea* [23]) and *Symploca*. Relative to their freshwater counterparts, toxins produced by marine cyanobacteria are thought not to present a direct health risk, mainly due to the fact that humans and domestic animals do not rely on seawater for drinking. However, they can lead to secondary health risks through bioaccumulation or poisoning of fishes and other seafood. To date, the major human health risk of marine cyanobacteria has been associated with members of the genera Oscillatoriales, *Moorea* and *Trichodesmium*. For example, *Lyngbya majuscula (Moorea producens)* is a prolific producer of diverse secondary metabolite compounds, including lyngbyatoxins and majusculamides. These marine cyanobacterial toxins have a broad range of biological activities, including dermatotoxic, cytotoxic, neurotoxic and tumorigenic activities [12,18].

Specific environmental conditions, especially enriched nutrient conditions, such as phosphorus and iron, promote the growth and formation of mats and coastal blooms attributed to *Lyngbya*/*Moorea* [26]. During these times, the overgrowth of the cyanobacteria and toxin production have become the cause for the closure of beaches partly due to the presence of skin irritant dermatoxin known to cause “swimmers’ itch” [27]. These outbreak events lead to reduced public confidence in seafood and equally damage the tourism industry. Estimates drawn in the U.S. state that harmful algal blooms (HABs) were costing approximately US$100 million per year to the U.S. economy in lost fishery production and stocks, human illness and lost tourism revenue [28], totalling upwards of US$1 billion during the past decades [29,30]. In Australia, the negative impact of cyanobacterial HABs was estimated to cost $180–240 million per year [31,32], with some blooms of photosynthetic microbes hypothesized to dramatically affect local businesses. Indeed, blooms of *Nodularia* and specifically *N. spumigena*, a brackish heterocystous genus producing hepatotoxin nodularin, have repeatedly caused issues around Australia and appear to be gradually expanding their biogeography [33,34]. In September 2008, one such bloom at a brackish lake in Queensland forced the closure for recreational access of a cable ski operation for a duration of three months at an estimated cost of AUD$300,000 [34].

Interestingly, the genome sequencing of *L. majuscula* (*Moorea producens*) suggested that it uses precursors from other surrounding bacteria to synthesise a proportion of its toxins [35,36,37]. Therefore, *Moorea’s* toxicity could be the result of a network association with metabolic exchanges between the various individuals in the microbial community. Thus, in order to mitigate the negative impacts of marine cyanobacterial toxins, it is important study these organisms within the appropriate ecological context.

## 3. Ecological Role of Marine Cyanobacterial Secondary Metabolites

Marine cyanobacteria can be found in various environmental niches, both as pelagic free-living forms and in the benthos, either forming mats on surfaces, or as symbionts of eukaryotes, such as sponges, ascidians or kelps. The benthic or host-associated forms of marine cyanobacteria appear to be a richer source of complex bioactive secondary metabolites, likely due to the character of this ecological niche, which facilitates a highly competitive and relatively nutrient-rich environment provided by the host [38,39]. Notably, multiple compounds, which were originally thought to be produced by higher organisms, such as sponges and ascidians, such as dolastatin and analogues (sea hare) leucamide A (sponge) and westiellamide (tunicate), are now shown to be synthesized by an associated cyanobacterium [12,37,40,41]. These marine cyanobacteria live in a complex ecosystem defined by close associations and intense competition from other members of the community and a higher frequency of encounters with numerous predators, including grazers and phage. Many of the metabolites they produce are thought to play an important part of defence mechanisms to attempt to gain the upper hand and thrive within their niche of choice.

Some marine cyanobacteria produce small molecules with structural similarity to compounds involved in bacterial quorum sensing, such as acyl-homoserine lactones [14]. These molecules act as inhibitors of bacterial quorum sensing; however, their mode of action is unclear, as, despite their structural similarity to known acyl-homoserine lactones, the cyanobacterial compounds were not shown to act as direct competitive inhibitors [42]. For example, *Lyngbya* (consisting probably of the renamed *Moorea* sp.) is known to proliferate in dense microbial mats and to produce several interfering metabolites, such as malyngamide, malyngolide and lyngbyoic acid [43,44,45,46]. In this habitat, the production of quorum sensing-interfering compounds may provide an advantage by interfering with regulatory networks of competitors [36]. Many of these compounds from marine organisms, including not only cyanobacteria, but also algae, fungi, tunicates and sponges (many secondary metabolites of which could be the result of cyanobacterial symbionts), have attracted commercial interest as they could prove useful in preventing marine biofouling through bacterial quorum sensing inhibition, as was shown for Microlins A and B from *L*. *majuscula* (now *Moorea* sp.) [47]. Planktonic marine cyanobacteria have also been reported to produce allelopathic compounds to gain advantage in some habitats. For example, *Synechococcus* CC9605, a coastal-dwelling cyanobacterium, has been shown to produce microcin C-like metabolites that inhibit the growth of other cyanobacteria strains [48], and marine *Cyanobium* strains produce bioactive compounds against a range of other marine organisms [49].

**Figure 2:**
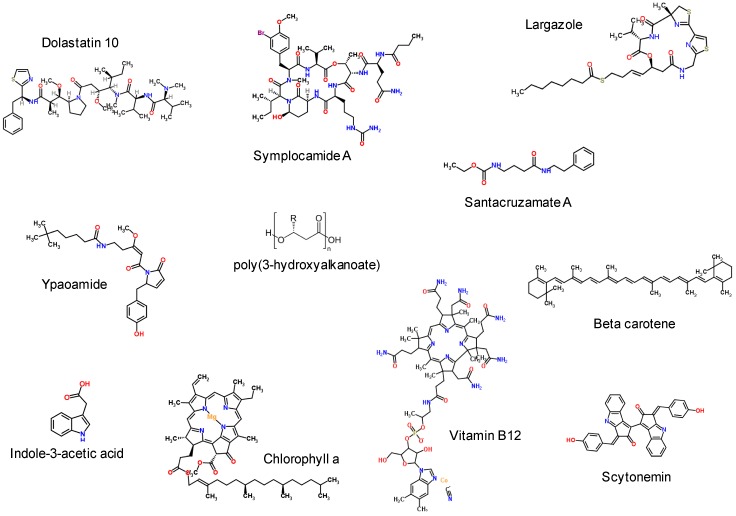
Examples of various secondary metabolites and pigments structures produced by marine cyanobacteria.

Due to their abundance and role as the base of the many aquatic food chains, cyanobacteria are constantly consumed by larger planktonic microbes, filter feeders and grazers. Hence, it is not surprising that cyanobacteria have developed effective chemical deterrents [36]. These molecules, which act as herbivore deterrents, are produced by benthic marine cyanobacteria and are excreted or exported to alter cell surface properties that lower their palatability to predators. Many have no demonstrated toxicity, but may act as repellents, leading to starvation of the grazer by removing their only food source, as these grazers will not feed on the cyanobacterial mat. For example, production of ypaoamide (Figure 2) by the assemblage of *Schizothrix calcicola* and *L. majuscula* acts a as deterrent to macrograzers, such as rabbitfish and sea urchins [50], and other yet unknown chemical deterrents from *L. majuscula* act against various grazers, such as sea urchins, crabs and other amphipods [51]. Despite these efficient deterrent mechanism, some mesograzers still feed on toxic cyanobacteria and have succeeded to adapt cyanobacterial defence systems for their own use. Indeed, sea hares accumulate large amounts of metabolites within their tissues, which are hypothesized to be derived from their cyanobacterial diet [52].

A different approach to surviving competition and predation employed by cyanobacteria is to establish a stable symbiosis with a higher organism, which provides shelter in return for nutrients and other compounds that benefit the host. For example, marine cyanobacteria exude up to 30% of their photosynthates, carbon-rich metabolites, into their surrounding environment [53]. This is hypothesized to be an important factor for their co-existence in symbiosis, e.g., in sponges, as this would alleviate the need of the host to rely solely on heterotrophy [54,55]. Multiple cyanobacteria produce auxin-like compounds, such as the phytohormone indole-3-acetic acid (IAA), which is hypothesized to be important in the establishment of cyanobacterial associations with photosynthetic eukaryotes (Figure 2). In support of this, Sergeeva *et al*. [56] found that 83% of the symbiotic isolates tested positive for the production of auxin-like compounds compared to 38% of the free-living ones. Moreover, IAA produced by *Nostoc* was recently shown to be necessary for it to colonize plant roots and additionally promoted plant growth [57,58].

Due to their obligate exposure to sunlight, cyanobacteria have developed mechanisms to protect their cellular components from the more harmful wavelengths of the light spectrum. Indeed, UV radiation induces damage at a number of cellular targets where damage can disrupt cell functioning. Some species produce photo-protective metabolites that offer a strong screen against ultra violet radiation, possess antioxidant properties [59,60] and can be stored inside or outside of the cell. Due to their UV-absorbing capacity, they are also referred to as cyanobacterial sunscreen [36]. Carotenoids, represented by a large and diverse set of compounds, including beta-carotene, zeaxanthin, echinenone and myxol pentosides (Figure 2) [61], are largely recognized as the most photo-protective of the intracellular molecules in cyanobacteria. Marine cyanobacteria also accumulate different variants of mycosporines and mycosporine-like amino acids (MAAs) that protect the cells mainly against UV-A radiation and, to a lesser extent, against UV-B radiation. Interestingly, the protective properties of MAAs can be transferred to higher trophic levels in the food chain, e.g., fishes were shown to contain various types of MAAs in their mucus, which may provide protection against UV radiation [62]. In addition to its UV protective role, MAAs can be modified by some species of sea hares and used in high concentrations as a chemical deterrent against competing species [63]. Some cyanobacteria also produce photo-protective molecules that are localized in the extracellular sheath, e.g., the UV-A protectant scytonemin (Figure 2). Scytonemin was also shown to protect the cell against UV-C and is believed to be an early form of photo-protectant [64].

Marine cyanobacteria are prolific producers of vitamin B complex and vitamin E [65]. They synthesize vitamin B12 for their internal metabolism and release excess through excretion in their surrounding environment. It was shown that nitrogen-fixing cyanobacteria excrete more vitamin B12, an N-rich molecule (Figure 2), than non-nitrogen-fixers [66]. Cyanobacteria have been hypothesized to be a major source of vitamin B12 that supports the growth of larger auxotrophic eukaryotic phytoplankton [66,67]. They may also support the growth of a range of auxotrophic bacteria and, hence, further sustain the diversity and health of the whole ecosystem (e.g., B12 auxotrophic SAR 11 bacteria) [68,69].

## 4. Biotechnological Applications for Marine Cyanobacterial Secondary Metabolites

### 4.1. Inhibitory Bioactive Metabolites

In addition to their ecological role, bioactive metabolites produced by marine cyanobacteria present valuable applications for agriculture, health and biofouling, but in particular, for the pharmaceutical industry, with potential for therapeutic applications [70].

Many cyanobacterial bioactive secondary metabolites are produced via non-ribosomal peptide synthase (NRPS) and polyketide synthase (PKS) biosynthetic pathways [15,71] compared to only a few derived from ribosomal proteins. Synthesized molecules include alkaloids, amides, fatty acids, indoles and peptides/depsipeptides [13,72]. This large spectrum of complex molecules presents great potential for therapeutic and biotechnological applications [13,73].

Due to the diminishing impact of classical antibiotics and the urgent need for new therapeutic drugs, there is a push for large programmes to screen secondary metabolites from marine sources for bioactive compounds [12,39,74]. Genes encoding novel bacteriocins [75] and antimicrobials, such as lantipeptides, have been discovered in numerous marine cyanobacterial genomes, e.g., *Prochlorococcus* MIT9313 [76], and are opening new avenues for research to combat multidrug-resistant microorganisms. The biocidal activity of cyanobacterial secondary metabolites towards biofilm-forming bacteria has also been proven to be of use for potential anti-biofouling applications, *i.e.*, deterrent of the fouling benthic diatom *Nitzschia pusilla* by Cyanobacteria from *Scytonema hofmanni* [77].

Numerous promising compounds have been identified with strong anti-proliferative activities and potent anti-cancer/tumour properties (e.g., apratoxins, dolastatin 10 (Figure 2) [78,79,80,81], as well as anti-viral (e.g., several anti-HIV compounds, as summarized in [10]), anti-malarial (e.g., gallinamide A) [82], anti-trypanosomal and anti-leishmanial activities (viridamide A, *Oscillatoria nigroviridis* [83]; dragonamide E [84]), in addition to anti-inflammatory and neurotoxic activities.

Some effective bioactive compounds have been shown to act as modulators of important metabolic enzymes. Several of these target or modulate, in a positive or negative manner, the activity of kinases or proteases associated with the development of tumours. While activators of protein kinase C, leading to tumour promotion, were isolated from the prolific metabolite producer *L. majuscula*, e.g., indole alkaloids, such as lyngbyatoxins [85]. Various other compounds of cyanobacterial origin and acting as enzyme inhibitors have been discovered in recent years. These include (serine-) protease inhibitors, lyngbyastatins [86,87], symplocamide, a chymotrypsin inhibitor with a high level of toxicity towards cancer cells (Figure 2) [88,89], and cyclodepsipeptides with elastase inhibitory activity, such as tiglicamides [90]. Some compounds have been shown to be highly specific histone deacetylases (HDAC) inhibitors, also affecting non-histone protein substrates and thus acting on downstream cellular pathways; altogether, they offer promising new disease treatments [91]. For example, two metabolites isolated from *Symploca* sp. were shown to have high activity towards HDAC: the potent and selective anti-proliferative santacruzamate A (Figure 2) [92] and the cyclic dipepsipeptide largazole specific to class I HDAC (Figure 2) [93,94,95,96].

Previous authors have reviewed the diverse molecules produced by both marine and freshwater cyanobacteria along with their structures and modes of action [12,42,73,97]. A large number of the compounds produced from either free-living or in symbiotic cyanobacteria are in clinical trials [72,73,79]. While a number of the trials (phase I and/or II) have been terminated due to strong host toxicity, e.g., the semi-synthetic cryptophycin 52, or cemadotin (LU103793), a synthetic analogue of dolastin 15 causing hypertension and cardiotoxicity [73], several of these natural products present promising leads for combinatorial chemistry and targeted modification techniques to develop efficient drugs with lower levels of toxicity, e.g., santacruzamate A and tasidotin (ILX-651, synthetic derivative of dolastatin 15) [73].

### 4.2. Nutritional Supplements, Pigments and Chromophores

As photosynthetic microorganisms, cyanobacteria harvest light as their energy source through a wide variety of photosynthetic antennae that are rich in pigments and chromophores. Several chromophores (e.g., tetrapyrroles) from the light-harvesting complexes (e.g., phycobilins and chlorophylls; Figure 2) have been reported to have beneficial health effects, e.g., providing micronutrients and macronutrients, aiding in digestion, *etc.* There is a blooming market for the use of cyanobacteria as beneficial human food/health supplements, and they are now being widely utilized in the nutraceutical industry. Among the most widely-used species is the halotolerant *Spirulina* (*Arthrospira platensis* and *Arthrospira maxima*). *Spirulina* cells have a high nutritional value and high digestibility, due to their richness in various nutrients and high protein content. They also present additional health benefits as a source of antioxidants, coenzymes and vitamins [59].

Marine phytoplankton, including cyanobacteria, are a rich source of pigments and carotenoids [61]. These have been historically used as colouring agents and colour enhancers and are now back to prominence due to health concerns over the use of chemical colouring agents.

Alongside their application as food for human consumption, microalgae are used as a feedstock in aquaculture and as soil additives for agriculture [98]. There is a drive to reduce the use of chemical fertilizers in agricultural soils due to their negative ecological impact. Seaweed has been traditionally used to improve soil quality in various regions of the world [99,100]. Cyanobacteria cells are rich in nitrogen, mainly from their N-rich photosystem antennae, with several species also fixing atmospheric dinitrogen; therefore, they are an ideal soil supplement already proven beneficial in the growth of rice [101]. Some cyanobacterial strains have the additional benefits of producing metabolites with herbicidal properties [102]. The production of compounds using cyanobacterial cells creates a large amount of biomass as a by-product that can be recycled as soil additives for agricultural purposes.

Cyanobacterial chromophores have equally found technical applications as molecular tools. These compounds, e.g., phycoerythrin, are routinely used as conjugates of molecules (antibodies) allowing qualitative and quantitative visualization from their desirable fluorescence properties with high signal intensity [103].

### 4.3. Biofuels, Industrial Processes and Engineering

Due to the depletion of Earth’s finite resources and the implications of climate change, there is currently a strong incentive to develop renewable alternatives to fossil fuels. Biofuels have been considered as a possible avenue to replace at least a proportion of the total fossil fuel consumption. Though the initial push focussed on biofuels produced from cellulosic waste, economic and technical production challenges with this approach have led to the emergence of algae-based biofuels (either from eukaryotic microalgae or cyanobacteria) as a more sustainable alternative [104]. The U.S. congress established a mandate, the Renewal Fuel Standard (RFS), requiring a proportion of the national fuel supply to be composed of alternative fuel sources, such as biofuel. The mandate required an increase of cellulosic biofuels, which is currently not being met. There are now increased discussions about the place of algae biofuel within the RFS [105,106]. Indeed, funding in algae R&D from the U.S. Department of Energy is currently on an increasing trajectory and has reached a cumulative $236 million as of December 2010. Recently (July 2015), the U.S. Department of Energy announced an $18 million USD grant on six new algal biofuel projects. Europe has equally seen a large push in algae R&D, in biofuel and other algal-derived products, to meet the European Union’s environmental regulations and in a conscious effort to reduce the global carbon footprint.

Many of the lipid-based biofuels are based on eukaryotic microalgae; however, marine cyanobacteria present further developmental advantages for optimizing industrial production, partly due to their smaller genome size, the fact that some of them are more genetically amenable and halotolerant [107], which provides the advantage of utilising abundant seawater resources for growth, rather than freshwater supplies, which are limited in many regions of the world. Several companies have made large investments in R&D over the past few years using modified brackish/marine cyanobacteria and are strengthening their aims towards commercial viability of their production process. Algenol (Fort Myers, FL, USA), founded in 2006, is using a modified cyanobacterium growing in saltwater (advantageous, as saltwater is a far more abundant and low cost resource than freshwater in many regions). The company produces various biofuels (ethanol, biodiesel, renewable gasoline and jet fuel), as well as high value chemicals [108]. Another successful, but secretive company and direct competitor of Algenol, is Joule Unlimited (Bedford, MA, USA) [109]. The Massachusetts-based company Joule Unlimited has been granted a U.S. patent (US 9,034,629B2) on 19 May 2015, covering their production process and genetically-modified cyanobacteria. The production from the brackish/marine cyanobacteria is not reliant on biomass production, but the compound is secreted into the culture medium. Furthermore, to suggest the commercial strength of the process, Joule Unlimited has established a partnership with Audi AG (September 2012) [110] and more recently (November 2015) merged with the biofuel maker company Red Rock Biofuels LLC (Fort Collins, CO, USA) [111]. Möllers *et al.* [112] also demonstrated that cyanobacterial biomass could be used as an efficient feedstock for bioethanol production by yeast fermentation. This study highlighted the high efficiency of transforming light energy to biomass, while also pointing out that cyanobacteria produce simplified cell walls and glycogen as the main storage polymer, which is far easier to mobilise than starch, the main storage polymer for eukaryotic algae.

Furthermore, some marine cyanobacteria, e.g., Cyanothece 51142, have been reported to produce high rates of hydrogen that can be harnessed as a renewable fuel [113,114]. Hydrogen is formed as a by-product of nitrogen fixation and is an attractive form of carbon-neutral renewable energy. The relatively high rates of hydrogen production found in nitrogen-fixing marine cyanobacterial provide promising avenues for further industrial applications.

Cyanobacteria and their metabolites are being exploited for use in wastewater treatment, bioremediation and biofouling. They have been reported to be an important part of the consortium for the oxidation of oil and complex organic compounds. However, it was shown that cyanobacteria were not directly involved in the process, but facilitated this process carried out by heterotrophic bacteria within the system [115]. Cyanobacteria were also shown to be prolific producers of exopolysaccharides (EPS), which have promising applications as biosurfactants and bioemulsifiers [116,117], e.g., emulcyan from the cyanobacterium *Phormidium* J1 [118]. EPS can equally be used for the absorption of heavy metals [99]. Biocide activities are of great interest for biofouling when applied directly to or mixed in paints for (submerged) surfaces [77].

The production of cyanobacterial biomass is inherently linked to CO_2_ sequestration; therefore, their use for industrial production has the potential to reduce the industrial carbon footprint. Some cyanobacterial strains assimilate and accumulate carbon into polymers, poly-hydroxyalkanoates (PHA) [10], which has attracted the attention of industrial companies as a way to potentially achieve carbon-neutral production of plastics in a much more cost-effective fashion compared to “traditional” plastics. These bioplastics also present a better biodegradability. Despite the efficient CO_2_ fixation, the production of biomass, in particular biofuels, requires additional nutrients, such as N and P. To alleviate the need for the input of extra nutrients, several applications have been established successfully using wastewater as a nutrient source [119].

### 4.4. Frontier Technologies

Photosynthetic organisms have the advantage of using light as an energy source and atmospheric CO_2_ as a carbon source. However, for both plants and the most commonly-used photosynthetic microbes, the demands for large spaces and copious amounts of freshwater pose challenges for keeping production costs low. The use of cyanobacteria growing in saltwater, with potential recycling of wastewater and use of non-arable land, would reduce both costs and environmental impacts [120]. Many isolates of marine cyanobacteria also display small streamlined genomes (<3.0 Mb) [121] and simplified cell-walls and storage polymers [112] relative to their eukaryote counterparts, which make them promising platforms for genetic and metabolic engineering to optimize the production of biomass, as well as yield and the recovery of secondary metabolites.

#### 4.4.1. Food Supplements

The production of high cyanobacterial biomass is a necessity for a wide range of applications, from small-scale setups to large-scale processes and, eventually, for commercialization. Cyanobacterial biomass production for food supplements continues to be a booming market, with continued expected growth [122,123,124]. One of the major cyanobacterial species utilised is the halotolerant *Arthrospira platensis* that can grow under freshwater, brackish and saltwater conditions [110]. Companies have based their business on the sale of *Spirulina,* grown in brackish waters, as a nutritional supplement for human consumption (protein, chlorophyll, vitamins and minerals) [125,126], e.g., Nutrex Hawaii (Cyanotech corporation, Kailua-Kona, HI, USA). The high nutritional value of cyanobacteria makes them attractive for the future of food. Several aeronautic agencies (NASA, National Aeronautics and Space Administration; JAXA, Japan Aerospace Exploration Agency; ESA, European Space Agency) are considering the use of algae to supplement astronauts’ diets. Furthermore, they have also launched research programs on the application of algae, such as *Spirulina*, as primary food for long-duration space travel or deep space missions. Indeed, in such missions, algae could be involved in replenishing oxygen, as well as providing a renewable source of food and fuel, while recycling waste [127].

#### 4.4.2. High Value Products

A promising pathway is the use of cyanobacteria by industries to produce cell biomass for the production of high value products. Hays and Ducat [128] proposed that cyanobacteria were ideal alternatives to plants for carbohydrate feedstocks. Proterro, Inc. (New York, NY, USA) uses a patented technology platform, modifying cyanobacteria that utilize waste CO_2_ to offer industrial- and food-grade sugars and a nutritional range of products, such as amino acids, nutrients and vitamins.

#### 4.4.3. Biofuels

Further developments linked to biomass production relate to the production of biofuels [129]. As previously discussed, the global push for cleaner energy and reduced carbon emissions has stimulated large-scale developments in order to decrease costs and streamline production. Several companies are investing in extensive R&D in this field. For example, Origin Clear Inc. (Los Angeles, CA, USA) [130] patented innovative processes for the separation of oil produced by eukaryotic microalgae, and they have refined a process for the live extraction of oil from microalgae (patent US 20120040428 A1). This technology allows for continuous oil production from high biomass compared to the batch-type production where the biomass is fully harvested before oil extraction.

In March 2016, the Helioculture process from Joule Unlimited, based on cyanobacteria, obtained approval from the U.S. Environmental Protection Agency (EPA) as a pathway for the generation of advanced biofuel RINs (D- code 5) under the RFS program [131]. It was calculated that this process achieves more than 85% lifecycle greenhouse gas reduction from the gasoline baseline. In addition to the secretion of bioethanol from the producing cyanobacterial cells, Joule Unlimited further processes its cyanobacterial biomass to produce an algal oil product. From the company’s successes, Joule Unlimited aims at constructing its first commercial facility by 2017, with a forecasted production price competitive to $50 per oil barrel.

#### 4.4.4. Energy-Efficient Green Buildings

**Figure 3:**
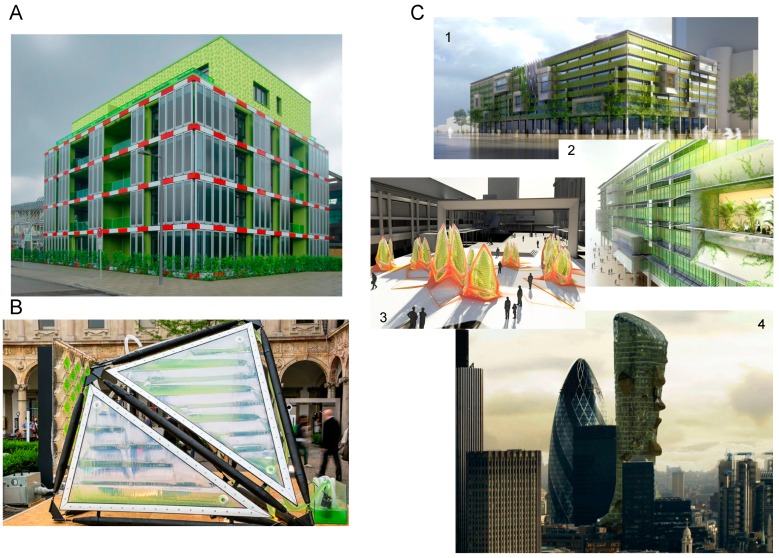
(**A**) Bio Intelligent Quotient (BIQ)—The Clever Treefrog—The Algaehouse, housing-project at the IBA Hamburg. Idea, concept and authorship: SPLITTERWERK, Label for Fine Arts, Graz; Arup GmbH, Berlin; B+G Ingenieure Bollinger und Grohmann GmbH, Frankfurt; Immosolar GmbH, Hamburg. Photo by SPLITTERWERK 2013. (**B**) Urban Algae Façade prototype by Cesare Griffa and Carlo Ratti Associati; prototyping team: Matteo Amela, Federico Borello, Marco Caprani; technical support by Environment Park Spa, Fotosintetica & Microbiologica Srl; lighting by iGuzzini. Photos by Filippo Ferraris. 2014 Salone del Mobile, Milan, Italy. (**C1**) Process Zero Exterior: the eight-story, 1960s-era building is among the 362 million square feet of office space the GSA must retrofit to reduce greenhouse gases by 30 percent before the 2020 deadline. Image credit: HOK/Vanderweil. (**C2**) Process Zero Exterior Facade Detail: algae, housed in glass tubes covering the building’s exterior, filters wastewater, consumes carbon dioxide from the nearby highway and uses photosynthesis to produce energy. Image credit: HOK/Vanderweil. (**C3**) Concept image created for the Perth Photobioreactor Design and copyright to Tom Wiscombe architecture. (**C4**) The FMSA Tower concept project, a self-sustainable skyscraper. Design and copyright to Dave Edwards. All photographs and artwork reproduced with permission of the authors.

Due to the move towards renewable energies and the reduction in carbon footprint, innovative concepts have emerged for the application of algae farms in setups other than traditional industrial productions. The idea of scalable algae farms incorporated into the landscape, both inside and outside of cities, has been proposed [132]. One of the most successful and sought after applications is the new concept of algae architecture as part of energy-efficient “green” buildings and landscape designs (Figure 3). For example, to integrate algae into the fabric of buildings for recycling CO_2_ and wastewater, thus providing a carbon-neutral workplace that manages its own wastes and transforms wastes into high value products, such as biofuels [133,134]. This concept has already been successfully applied, for example in the Bio Intelligent Quotient (BIQ) house in Hamburg (Figure 3A) constructed and finished in 2013 [135]. The BIQ house is surrounded by facades of microalgae cultivation panels linked and integrated into the functioning of the building to reduce its environmental carbon footprint. The heat generated from the algae growth is recycled through thermal exchangers and used for heating, as well as producing biomass for use in biodiesel [135]. Other examples of integrated algae design can be found in a retrofitted system on the La Defense building in Paris, France, aimed to reduce the environmental footprint of the building merging concepts of algae farms, algae biofuel and wastewater treatment [136], and in Italy, where the company Algaetecture presented a prototype urban algae canopy (Figure 3B) [137]. Cesare Griffa Architecture lab [138] designed several projects based on microalgae systems, e.g., Bioskin and the Lillies series (part of the prototype urban algae canopy; Figure 3B), that can be adapted to and used with various microalgae and, in particular, *Spirulina* sp. Several eco-architects have further worked on integrating living plant/algae within architecture prototypes exploiting the production of microalgal biomass into the functioning of the building design (Figure 3C).

#### 4.4.5. Genetics and Synthetic Biology

The evolution of cyanobacterial strains for growth at high densities and for enhanced production levels of desired metabolites would be essential in the large-scale industrial applications with an overarching aim of achieving optimal production at reduced costs [139]. The past decade has witnessed a boom in the sequencing of the genetic material of microorganisms and a revolution in omics technologies. The emphasis has moved from the simple analysis of one gene in one system to global analyses of biological networks and synthetic redesign of biological systems. The interdisciplinary field of synthetic biology provides interesting options for future biotechnological applications.

The development of -omics technologies and the large amount of sequencing data being generated from previously untapped habitats and microbial communities, provide a great resource for potential new targets/biosynthetic pathways [37,140,141]. Genetic engineering for the synthesis of new molecules is already successfully conducted in *Escherichia coli*. For example, patellamide, originally identified from a cyanobacterial symbiont of ascidians, was successfully produced by recombinant *Escherichia coli* [142]. Cyanobacteria have already been modified to produce metabolites through synthetic pathways, such as isopropanol [143]. Using identified biosynthetic genes (PKS/NRPKS) in order to engineer complex molecules with specific bioactivities is a widespread approach in combinatorial chemistry and/or synthetic biology [144,145]. These developments open infinite possibilities in the development and assessment of lead compounds in a widening range of applications, including in the development of pharmaceuticals.

## 5. Conclusions

Since much of the attention in relation to metabolite production has been historically focused on their freshwater counterparts, marine cyanobacteria present a relatively untapped resource in terms of evolutionary diversity and industrial potential. They are prolific producers of diverse and complex secondary metabolites with potential applications in health, biofuels and bioengineering. They have minimal genomes and low cellular resource requirements, which make them well suited for genetic and metabolic engineering. In light of demands on natural resources, including freshwater, nutrients and arable land, marine cyanobacteria offer an important advantage over their freshwater counterparts for industrial-scale processes, *i.e.*, they are adapted to growing in brackish and salt water. Coupled with their ability to convert sunlight to energy, these organisms have the capacity to serve as low cost, adaptable cellular factories capable of producing high-value products and biofuels with low environmental impact.

Cyanobacteria and, particularly, the marine dwellers have become increasingly integral parts of future innovative projects, from aeronautic programmes to concept projects in sustainable architecture. The incorporation of algae into novel architectural designs has the potential to improve waste recycling, climate control and reduce the carbon footprint of commercial buildings. Much is still unknown about marine cyanobacterial metabolites; however, there is a great deal of progress being made using recent advances in molecular techniques, including large-scale environmental genome sequencing projects, metabolic modelling and synthetic biology approaches. Expanding the potential biotechnological benefits of marine cyanobacteria will benefit from collaborations across the fields of ecology, genomics, chemistry, health research and engineering and will result in the development of new technologies, including extending the range of cyanobacterial metabolites beyond traditional uses, optimizing biofuel production by using non-arable land and abundant saline water resources and contributing to the ecological buildings of the future.

## Abbreviations

The following abbreviations are used in this manuscript:

CO_2_	carbon dioxide
FMSA	financial market service authority
GSA	general services administration
HIV	human immunodeficiency virus
HOK	formerly Hellmuth, Obata + Kassabaum, design firm
IBA	International Building Exhibition
N	nitrogen
P	phosphorus
R&D	research and development
U.S.	United States (of America)
UV	ultra-violet
